# Supplementary material for: The Prognostic Value of Neutrophil-to-Lymphocyte Ratio and Platelet-to-Lymphocyte Ratio in Patients with Hepatocellular Carcinoma Receiving Atezolizumab Plus Bevacizumab
Source: Cancers (Basel). 2022 Jan 11;14(2):343. doi: 10.3390/cancers14020343 (PMC8774110; doi:10.3390/cancers14020343)
Supplement: Supplementary file 1 [file cancers-14-00343-s001.zip › cancers-1523402-supplementary.pdf]

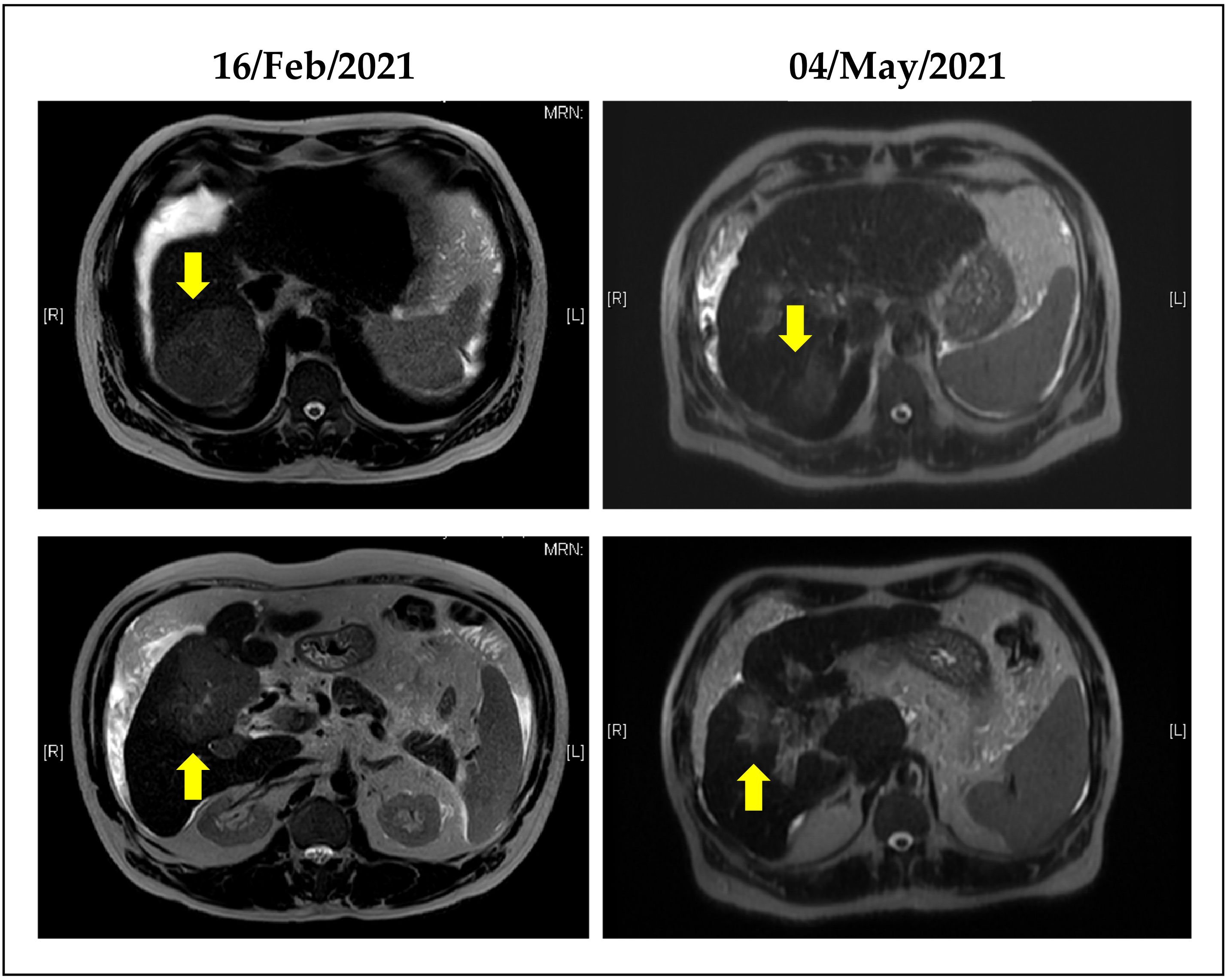

Figure S1. The 53-year-old man had past history of hepatitis B and liver cirrhosis, and has been diagnosed with hepatocellular carcinoma in Feb/2021. Magnetic resonance imaging (MRI) of liver revealed multiple liver tumors (arrow) over S5/S7/S8 with left, right and main portal vein thrombosis. Then he received atezolizumab plus bevacizumab since Feb/2021; image was followed after completion of three cycles of this combination therapy. MRI of liver demonstrated that decreased in size of liver tumors and portal vein thrombosis, indicating **partial response (PR)**.

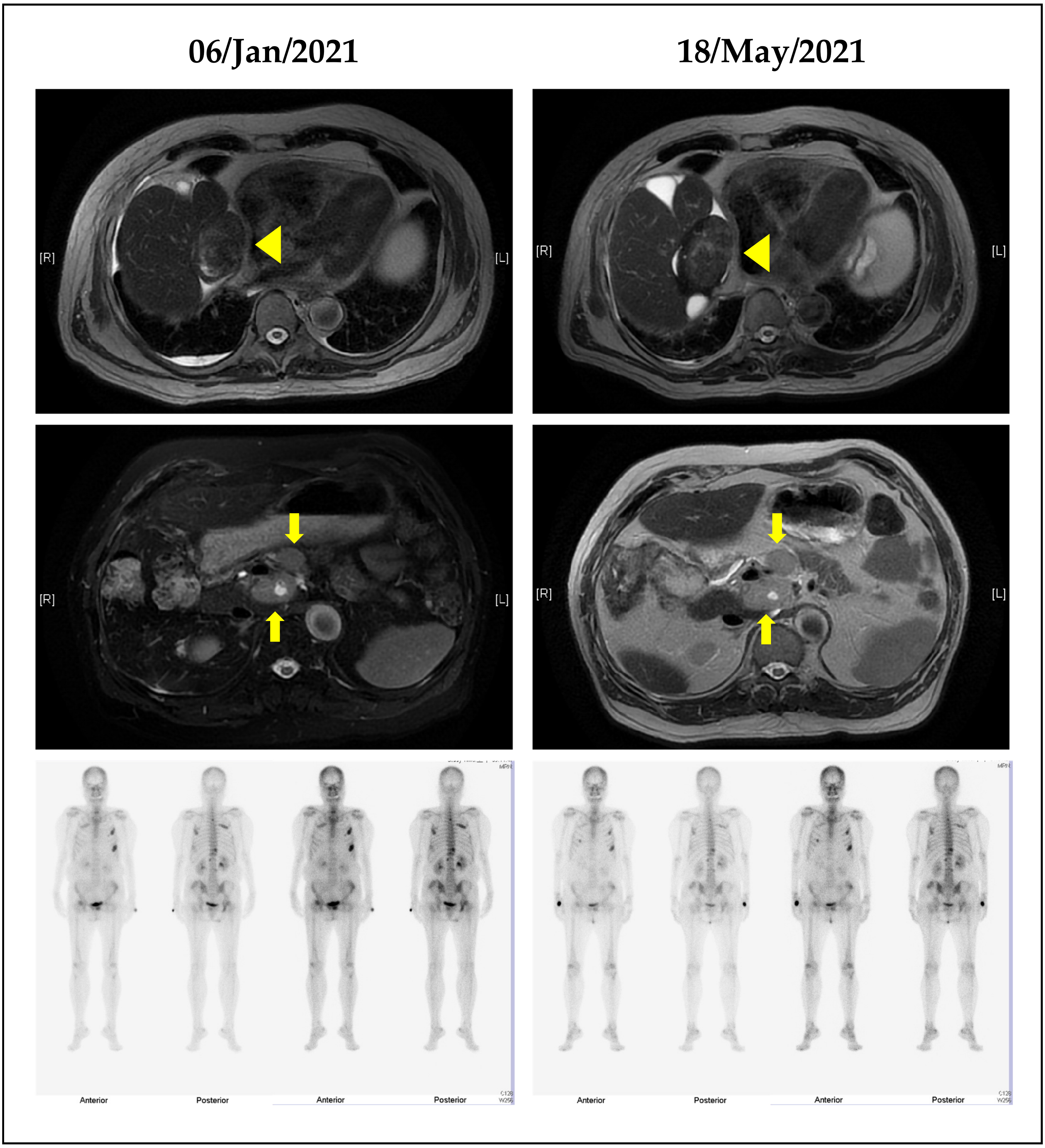

Figure S2. The 61-year-old woman has been hepatitis B related liver cirrhosis and splenomegaly, and hepatocellular carcinoma was diagnosed in Jan/2021. Magnetic resonance imaging (MRI) of liver showed liver tumors (arrowhead) over S4/S7/S8 and enlarged lymph nodes (arrow) over gastrohepatic and precaval regions. In addition, multiple bone metastasis was detected by bone scan. After that, she received atezolizumab plus bevacizumab for 5 cycles, and stable condition of liver tumors was mentioned by followed MRI of liver.

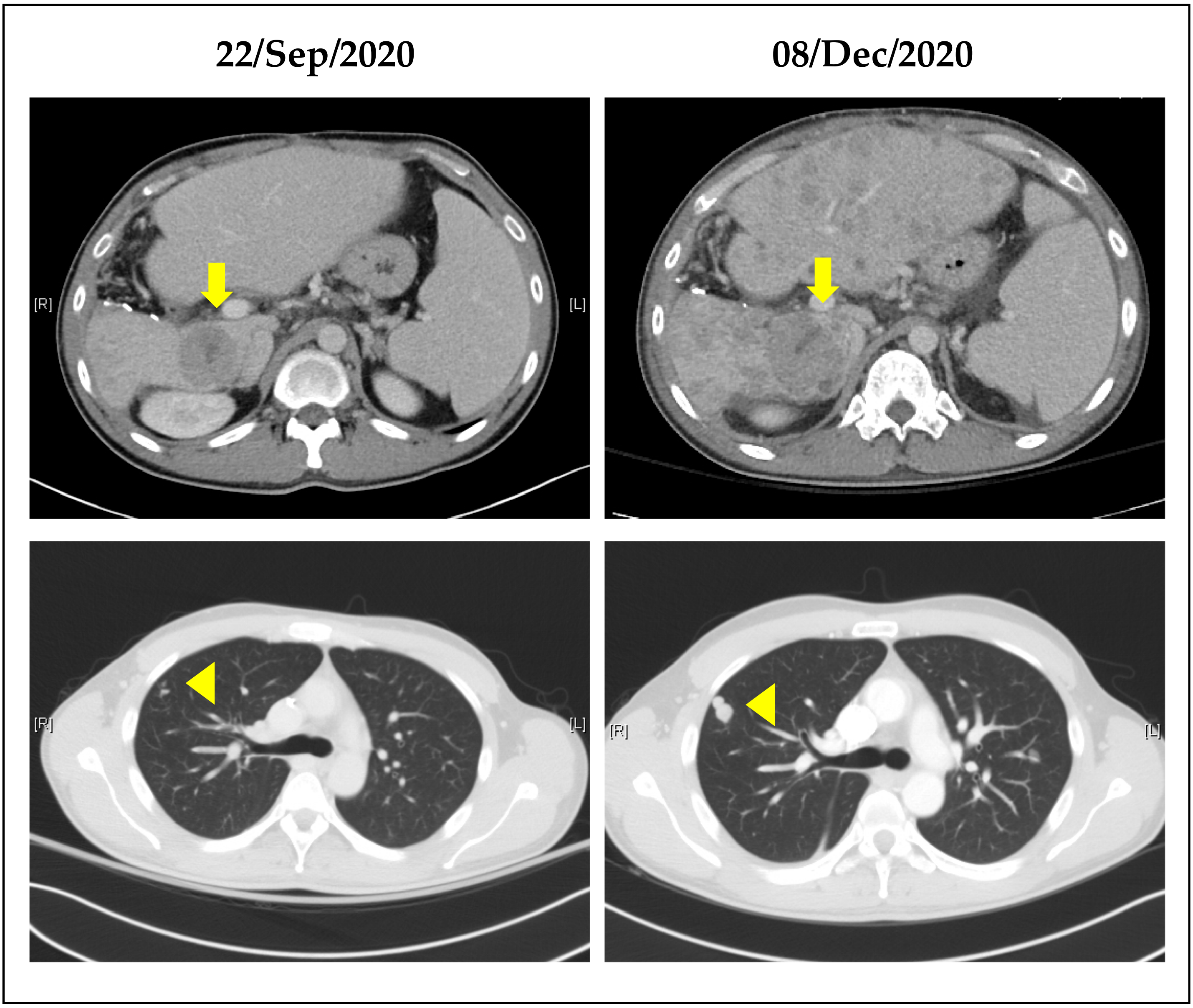

Bone scan also revealed mild regressive change of bone metastasis.

The treatment response was regarded as **stable disease (SD)**.

Figure S3. The 31-year-old man has been diagnosed with hepatocellular carcinoma with lung metastasis in Sep/2020. Computed tomography (CT) of liver showed liver tumor over S7 (arrow) and CT of chest revealed one small nodule (arrowhead) over right upper lobe. Atezolizumab plus bevacizumab were given since Sep/2020 with a total of three cycles. Followed CT of liver demonstrated increased in size of liver tumor over S7, accompanied with multiple new liver nodules over both lobes, suggesting progression. On the other hand, markedly progressive change of metastatic lung nodule over right upper lobe was also mentioned on CT of chest. In conclusion, the response to atezolizumab plus bevacizumab was **progressive disease (PD)**.
